# Next-Generation Sequencing in Clinical Molecular Diagnostics of Cancer: Advantages and Challenges

**DOI:** 10.3390/cancers7040874

**Published:** 2015-10-14

**Authors:** Rajyalakshmi Luthra, Hui Chen, Sinchita Roy-Chowdhuri, R. Rajesh Singh

**Affiliations:** 1 Department of Hematopathology, The University of Texas MD Anderson Cancer Center, 8515 Fannin Street, Houston, TX 77054, USA; rluthra@mdanderson.org; 2 Department of Pathology, The University of Texas MD Anderson Cancer Center, 1515 Holcombe Blvd, Houston, TX-77030, USA; hchen7@mdanderson.org (H.C.); SRoy2@mdanderson.org (S.R.-C.)

**Keywords:** cancer genomics, next-generation sequencing, guidelines, molecular diagnostics, mutation

## Abstract

The application of next-generation sequencing (NGS) to characterize cancer genomes has resulted in the discovery of numerous genetic markers. Consequently, the number of markers that warrant routine screening in molecular diagnostic laboratories, often from limited tumor material, has increased. This increased demand has been difficult to manage by traditional low- and/or medium-throughput sequencing platforms. Massively parallel sequencing capabilities of NGS provide a much-needed alternative for mutation screening in multiple genes with a single low investment of DNA. However, implementation of NGS technologies, most of which are for research use only (RUO), in a diagnostic laboratory, needs extensive validation in order to establish Clinical Laboratory Improvement Amendments (CLIA) and College of American Pathologists (CAP)-compliant performance characteristics. Here, we have reviewed approaches for validation of NGS technology for routine screening of tumors. We discuss the criteria for selecting gene markers to include in the NGS panel and the deciding factors for selecting target capture approaches and sequencing platforms. We also discuss challenges in result reporting, storage and retrieval of the voluminous sequencing data and the future potential of clinical NGS.

## 1. Introduction

Characterizing genomic aberrations in tumors for predictive and prognostic purposes by genome sequencing has become an integral part of the precision medicine approach [1,2,3,4,5,6]. Until recently, most of the techniques used for this purpose included low- and medium-throughput traditional techniques such as Sanger sequencing, pyrosequencing, allele-specific polymerase chain reaction (PCR), high-resolution melt curve analysis, fragment analysis, and primer-extension coupled with mass spectroscopy. However, the increased discovery rate of clinically relevant biomarkers as a result of extensive characterization of cancer genomes has necessitated the testing of multiple genes per tumor as the standard-of-care. The aforementioned traditional technologies are unable to meet this demand. The revolutionary second- or next-generation sequencing (NGS) technologies provide a viable alternative because of their massively parallel sequencing capability, which enables the simultaneous screening of multiple genes in multiple samples [7,8]. In spite of the obvious advantages of the NGS technologies, their successful implementation for routine molecular screening of tumors is challenging because of the complexities of the assay and the need for thorough validation before implementation in molecular diagnostic laboratories. Here we discuss the advantages and the potential of these revolutionary technologies and the associated challenges.

Compared with earlier genome characterization techniques, NGS technologies possess distinct advantages more suitable to addressing the challenges associated with the increasing demands for testing of multiple gene markers with lower inputs of nucleic acids. The foremost advantage of the NGS technologies is the massively parallel sequencing capability. For routine tumor sequencing, this feature facilitates simultaneous sequencing of multiple targeted genomic regions in multiple samples in the same run. This is a very important advantage that enables screening of large numbers of samples while keeping short turnaround time for timely clinical reporting.

More importantly, screening multiple markers with NGS technology requires a single input of relatively low-quantity DNA or RNA in contrast to traditional sequencing technologies, which need cumulatively larger quantities of input nucleic acid. This also decreases the overall cost of multiple-marker screening compared with the costs of low- and medium-throughput platforms. An example of direct cost comparison for a 50 gene NGS test and several single-gene tests using traditional sequencing techniques is provided in Supplementary Table S1. In addition, NGS is able to provide simultaneous screening of a variety of genomic aberrations such as single-nucleotide variants (SNVs), multiple-nucleotide variants (MNVs), small and large insertions and deletions, and copy number variation (CNVs) of the genes. In NGS, on average, the targeted areas of interest are repeatedly sequenced hundreds and even thousands of times, providing high sensitivity and confidence for mutation detection. NGS is also quantitative as the allelic fraction of the mutation can be gleaned by the number of DNA strands with mutation in the background of strands with the wild-type sequence. These considerable advantages make NGS very desirable for routine clinical sequencing of tumors; however, implementation of NGS poses several challenges, which we discuss below.

### 1.1. Performance Validation

NGS technologies encompass several different approaches and platforms that perform high-throughput massively parallel sequencing. Traditionally, all of the NGS technologies were used for research purposes only. Integrating NGS into a clinical diagnostic setting requires thorough validation with respect to consistent performance and accuracy, as per the stringent regulations and guidelines established by the regulatory agencies governing the clinical laboratories [9]. However, until recently, these guidelines were defined only for low-throughput assays and therefore had to be reoriented and reinterpreted to deal with the high complexity and capacities of NGS. Recently, the U.S. Centers for Disease Control and Prevention (CDC), American College of Medical Genetics and Genomics (ACMG), Association for Molecular Pathology (AMP) and College of American Pathologists (CAP) developed guidelines defining the approaches for effective validation and implementation of NGS technologies and meaningful reporting of NGS results [10,11,12,13]. Furthermore, several clinical laboratories also recently published their approaches and experiences in validating and implementing various NGS platforms [14,15,16,17,18,19,20]. Table 1 includes a list of assay performance parameters that need to be established for general molecular and NGS tests [9,13].

The overall approach for the validation includes sequencing a set of clinical tumor specimens with known somatic aberrations as detected by a validated orthogonal sequencing platform in the laboratory. These specimens need to harbor clinically reported mutations in genes-of-interest and variant types of interest (SNVs, MNVs, insertions and deletions, and gene copy number variations), to ensure adequate validation. In addition to clinical specimens, well characterized human cell lines positive for somatic mutations (NCI-60) and germ line polymorphisms (HAPMAP-Haplotype Map) can be used [21,22]. The HAPMAP cell lines from Coriell Institute serve as valuable reference materials with common variants in human genome mapped. They are being brought to prominence by initiatives such as the Genome in a Bottle Consortium (GIAB), spearheaded by the National Institute of Standards and Technology (NIST). One such extensively characterized cell line (NA12878) was released recently by NIST as a reference sample [23]. These cell lines represent a very valuable resource of well-characterized genomic variants to establish the sensitivity and specificity of the NGS assays. However, care must be taken to process these cell lines to mimic the clinical samples for which the NGS assay is being validated. For example, if the NGS assay is for solid tumors, for which the majority of samples are expected to be formalin-fixed and paraffin-embedded (FFPE), cell lines used for validation must also be FFPE to ensure that the nucleic acids isolated from these cells simulate the compromised quality of nucleic acids from the FFPE tissues [18,19]. 

Currently, companies like Horizon Diagnostics (Cambridge, UK) and Acrometrix (Fremont, CA, USA) offer commercially available synthetic standards which can be used as controls for NGS assays. Horizon Diagnostics engineers cell lines to introduce mutations of interest in the genome at desirable variant allelic frequencies. This approach can be used to generate reference standards for any gene and type of variant. Hence these standards are proving to be a valuable resource, especially for genes with low natural mutation frequency. Engineering mutations into the genome of the cell lines not only simulates the mutational burden in tissue but also alleviates the problem of contamination associated with using DNA oligomers or plasmids as alternative reference standards. Acrometrix offers DNA controls with a variety of variants in multiple cancer-related genes prepared by blending synthetic and genomic DNA, which can be used as a valuable control for multiple NGS assays and platforms. Compared to patient samples, which are often limited, synthetic and cell line controls provide ample nucleic acid material that can be used to establish multiple assay performance parameters and ensure continued assay quality and accuracy.

**Table 1:** Assay performance parameters. The recommended assay parameters to be established for validation and implementation of NGS assays in a molecular diagnostic laboratory are listed (summarized from [9,13]).

Performance Parameters	Explanation	Purpose
Analytical Sensitivity*	Portion of samples in the validation set that are positive for mutations, as detected by a validated platform, and are correctly identified as positive	Ability of the assay to detect true sequence variants (false-negative rate)
***Analytical Specificity***	Portion of samples in the validation set that are negative for mutations, as established by a validated platform, and are accurately classified as negative	Probability of the assay to not detect mutations where none are present (false-positive rate)
***Accuracy***	Concordance between the genomic sequences obtained by the NGS assay and the reference sequence	Measure of sequencing accuracy and error rates
***Precision***	The tendency of achieving accurate results regarding detection of mutations across users and sequencing runs	Measure of reproducibility of mutation detection by the assay and inter-user reproducibility
*Limit-of-detection* *	The lower limit of mutation detection	To establish the detection limit for different variants such SNVs, MNVs, insertions, deletions, CNVs, and gene fusions
***Sequencing depth and allelic frequency cutoffs***	Define the minimum sequencing coverage necessary for confident detection and calling of variants	Needs to be established for different variants such as SNVs, MNVs, insertions, deletions, CNVs, and gene fusions

* Some of the parameters can have variable definitions or sometimes used interchangeably. For example, *analytical sensitivity* is also defined as the ability to detect limited amounts of an analyte, which overlaps with the *limit-of-detection* definition in the table.

A comparison of the analytical sensitivity of NGS assays observed in our laboratory to the orthogonal sequencing platforms has been provided in Supplementary Table S2. During validation of an NGS test, it is also desirable to cross-validate with an already established NGS platform in the laboratory, preferably one that uses a different sequence capture and sequencing chemistry [17,18]. This provides a one-on-one comparison of sequencing large genomic areas interrogated by NGS and provides better confidence of validation. Such comparisons are not possible using traditional low- and medium-throughput sequencing platforms, which usually sequence small and focused areas of the genome. 

### 1.2. Choices and Challenges of NGS Technology

In contrast to traditional sequencing technologies, NGS is capable of massively parallel sequencing of the genome. However, similar to the earlier technologies, majority of NGS platforms use the sequencing-by-synthesis (SBS) for sequencing [24]. In this approach, the DNA strand to be sequenced is used as a template, and a complementary strand is synthesized, during which the sequence of the template strand is obtained. The most common method uses four distinct fluorescently labeled nucleotides and optical imaging to visualize the growing complementary strand (as in the case of MiSeq and HiSeq Illumina Sequencers). This is referred to as 4-channel sequencing, as all fluorescent tags have to be imaged for sequencing [24,25]. A recently introduced NGS sequencer from Illumina (NextSeq) employs a novel approach in which three fluorescently labeled nucleotides (C, T, and A) and one unlabeled nucleotide (G) are used for sequencing. Filters are used for 2-channel imaging, which distinctly detects C bases (red fluorescence) and T bases (green fluorescence). The A bases are labelled by both green and red fluorescent tags which appears as yellow fluorescence with both filters. Lack of any fluorescent signal is considered a G base incorporation. Optical detection is also used in the sequencer from Pacific Biosciences, in which distinctly labeled nucleotides held by the DNA polymerase prior to incorporation are imaged during SBS to obtain the sequence of the template [26,27].

In addition to these technologies, several non-optical technologies of genome sequencing have been reported, including the Ion Torrent semiconductor–based sequencing technology, which has gained considerable acceptance [28]. The Ion Torrent semiconductor–based technology also uses SBS, where sequencing is performed in microscopic wells interfaced with a semiconductor chip. The DNA of interest is clonally amplified on microscopic beads and unlabeled nucleotides are introduced in a predetermined order one at a time. Upon incorporation, the protons released from the 3′-OH group during formation of the phosphodiester bonds results in a change in pH, which is measured by the semiconductor chip. Validation and implementation of this technology for numerous research and clinical applications has been reported [18,19,29]. Also, a non-SBS and non-optical technology of sequencing, which is referred to as the Nanopore technology (Oxford Nanopore Technologies, Oxford, UK), has been described in which single strands of nucleic acids are transported through a protein nanopore by application of an electric field. The movement of the nucleotides through the pore results in distinct modulation of the electric field, which is characteristic of each nucleotide and helps in gleaning the sequence. Compared with the aforementioned sequencing technologies, the Nanopore technology has major advantages, including limited pre-sequencing preparation, a small foot-print, flexible run times, and long reads, but has limitations due to high error rates [30,31,32]. A summary of the most commonly used NGS sequencers, their underlying technologies and their capabilities has been provided in Table 2.

**Table 2:** NGS sequencers and sequencing technology. Prominent NGS platforms, the sequencing technology, features and capabilities have been summarized.

Company	Sequencer	Sequencing Technology	Comments
**Illumina Inc**	MiSeq HiSeq NextSeq	Sequencing-by-Synthesis (Reversible terminator-Based)	Optical Sequencing MiSeq and HiSeq use a mixture of 4 fluorescently labelled nucleotides for SBS (4 channel imaging) NextSeq uses 3 fluorescently labelled nucleotides (C, T and A) and G is unlabeled (2 channel sequencing) Chain termination and imaging at each cycle of SBS ensures high sequencing quality at homopolymer and repeat regions Clonal amplification of template by bridge amplification on the surface of glass flow cell Paired-end sequencing ensures high confidence of mutation detection Read length—75–600 bp **Capability**—targeted sequencing, whole exome, transcriptome and whole genome
**Life Technologies**	Ion Torrent Personal Genome Machine (PGM) Ion Proton	Sequencing-by-Synthesis (Semiconductor-based)	Revolutionary non-optical semi-conductor sequencing Unmodified nucleotides Introduced individually during SBS Clonal amplification on microscopic beads Nucleotide incorporation detected by measuring the change of pH due to release of H^+^ from the 3′-OH group during nucleotide incorporation Readlength—200 bp and 400 bp High false positive rates at homopolymer areas **Capability**—targeted sequencing, whole exome and trascriptome
**Pacific Biosciences**	PACBIO RSII	Single molecule realtime (SMRT) sequencing	Optical sequencing Mixture of 4 distinctly labelled nucleotide used for incorporation Longest reads of any NGS sequencer (upto 40,000 bp) Sample amplification is optional eliminating amplification and GC bias **Capability**—Targeted sequencing, whole exome, transcriptome and whole genome Long read capability is very useful for *de novo* genome assembly

## 2. Choices for NGS Panel Content and Target Capture Technology

For the purpose of routine screening of clinical samples, sequencing selective markers of established clinical significance provides the most efficient approach. In this respect, while designing a panel it is important to consider the clinical importance of the genes to be included and the targeted area in the genes to be sequenced. One can choose commercially available panels or have them custom-designed per the requirement. Several commercial predesigned gene panels are available that can be validated and implemented in diagnostic laboratories [14,18,19]. In some cases, the predesigned panels could be further customized by the inclusion of additional gene markers that the laboratory deems important [17].

However, the predesigned panels could also have markers that may not be relevant to the tumor type being tested by the laboratory, resulting in the waste of sequencing real estate and higher costs. To circumvent this and to provide more flexibility, most companies also have relatively smaller tumor-specific panels to cater screening of specific genes relevant to the tumors type. Furthermore, custom panels can be designed, over which the laboratory has complete flexibility, not only over the selection of genes but also over the areas in the genes to be sequenced (most frequently mutated hotspots or entire exons in the gene). Considerable care must be taken before the selection of the genes and the areas that are to be included for the panel design. Since most of the target capture technologies include the multiplexed probe or primer-based target capture, once the panel is designed and validated, the addition or removal of markers to a panel could mean redesign and revalidation of the panel before implementation.

Several methods are available for selective enrichment of targeted genomic areas for NGS. The selection of these methods is crucial for NGS and is determined by several factors such as the sample type (fresh, frozen, or FFPE), quantity and quality of DNA or RNA routinely available. For instance, peripheral blood and bone marrow samples from patients with hematological malignancies yield relatively large amounts of high-quality DNA and RNA; in contrast, the samples from solid tumors, which are predominantly FFPE samples, yield lower quantities of nucleic acids with compromised quality. Hence for FFPE samples, high multiplexed PCR is the most preferred methodology for targeted enrichment and genomic library preparation. This approach is used by the Ion AmpliSeq (ThermoFisher, Grand Island, NY, USA) and GeneRead target capture technology (Qiagen, Valencia, CA, USA), which can effectively amplify targeted areas of interest for sequencing from as low 10–40 ng of FFPE DNA. In contrast, for fresh or frozen tissue samples, in addition to the PCR-based enrichment, probe-hybridization–based capture can also be used where probes specific to genomic areas of interest are hybridized followed by target capture and amplification. Several methods are available for probe-based capture technologies such as TruSeq and Nextera (Illumina Inc, San Diego, CA, USA), SureSelect and Haloplex (Agilent Technologies, Santa Clara, CA, USA), and Xgen (Integrated DNA Technologies, San Jose, CA, USA) target capture technologies. Studies that provide in-depth comparisons of these methods are valuable resources to make the most suitable target capture method selection [33,34,35]. A summary of the various enrichment technologies for NGS has been provided in Table 3. 

**Table 3:** Target enrichment methods and systems for NGS. A summary of different target enrich approaches for NGS, the enrichmentapproach and the required DNA input as per the manufacturer’s recommendation.

Company	Enrichment Technology	Enrichment Approach	Options, recommended DNA input and comments
**Illumina Inc**	TruSeq	DNA probe-based capture	TruSeq Amlicon Kit—(50 ng–250 ng high quality DNA, 250 ng FFPE DNA)
			TruSeq DNA PCR free (Low Throughput)—(1 µg)
			TruSeq DNA PCR free (High Throughput)—(2 µg)
			TruSeq NANO Low and High throughput kit (LT and HT)—(100–200 ng)
			NeoPrep System—Automated enrichment and library preparation system
**Life Technologies**	AmpliSeq	PCR-based amplification	10 ng DNA per primer pool ( up to 6000 primers)
			Well-suited for low quantity and quality DNA samples like FFPE samples
**Agilent Technologies**	SureSelect	Hybridization and capture using cRNA-baits	200 ng–3 µg DNA input
	Haloplex	Restriction enzyme digested DNA used as template	200 ng DNA input
		Circularization probe-based target enrichment	
**Qiagen**	GeneRead	PCR-based amplification	40 ng DNA input
**Integrated DNA Technologies**	Xgen Lockdown probes	DNA probe-based capture	500 ng DNA input
**RainDance Technologies**	ThunderStorm and ThunderBolt systems	Droplet PCR-based amplification	ThunderBolt system 10–50 ng for limited gene panel size.
			ThunderStorm system 500 ng–1 µg depending on the gene panel size

## 3. NGS Data Analysis and Clinical Reporting

Every NGS run typically includes parallel sequencing of multiple genomic areas in several barcoded and multiplexed samples. Typically, the NGS data analysis pipeline includes base-calling as the first step, in which the base sequence is assigned using the signal read-out. This could be optical measurement of the fluorescent tags on nucleotides (Illumina and PacBio) or measurement of pH change (Ion Torrent). Base-calling is followed by alignment of the sequence reads to a reference genome. Generally for targeted sequencing, the targeted areas-of-interest in the genome are specified for alignment to simplify the alignment process. Different platforms prefer distinct alignment algorithms suitable for their sequencing output. For instance the MiSeq Reporter software uses the Burrows-Wheeler Aligner (BWA) [36] and Torrent Suite software on Ion Torrent–PGM and Ion Proton uses the Torrent Mapping Alignment Program (TMAP) [29]. Sequence alignment results in filtering and elimination of off-target reads and comparison of the on-target reads to identify genomic aberrations. This is referred to as *variant calling*, and the aberrations could be simple single or multi nucleotide variants (SNVs and MNVs) or more complicated variations such as small and large insertions and deletions, gene copy number variants (CNVs) or gene fusions. Accurate identification of the complex genomic variants could be challenging; making the choice of an appropriate variant-calling algorithm crucial. To cover the entire range, multiple variant-calling algorithms may be used, especially for complex insertion-deletions, CNVs and gene fusions [37,38,39].

The management of high volumes of data generated by NGS is also a challenge for clinical laboratories, which are generally used to low- or medium-throughput tests. NGS data needs to be stored so as to provide complete traceability of the results, with records of various versions of software and algorithms in compliance with the guidelines from regulatory agencies. With multiple steps in NGS data processing, multiple file formats are generated such as FASTQ (base calling and quality scores), BAM, SAM (post-alignment information), and VCF (variant calls). As NGS analysis ranges from small to large gene panels and from whole exome to whole genome analysis, it is impractical to store all files generated in this process. Generally, files that possess appropriate information to repeat the analysis are sufficient as per the guidelines from the regulatory agencies [10,11].

Variant calling generally results in numerous variant calls which in addition to genuine calls also could include spurious calls (sequencing artifacts, errors caused by repetitive sequences such as tandem repeats and homopolymers). Filtering spurious mutations for clinical interpretation and reporting represents a major interpretation challenge. For this purpose, the variant calls are filtered according to multiple criteria established by the laboratory during assay validation. This generally includes considering adequate sequencing quality, sequencing depth, allelic frequency, correlation with tumor percentage, and presence of the variant in both forward and reverse sequencing reads or lack of strand bias. Direct visualization of the sequence reads, using tools such as Integrated Genome Viewer (IGV) [40] or UCS genome Browser [41], is also a very important part in establishing the authenticity of the variant before reporting. A summary of parameters used for quality control at each step of clinical NGS has been provided in Table 4.

**Table 4:** Quality control (QC) metrics for NGS. The quality control metrics used at different steps in the NGS workflow, the indicators, measurement methods and their significance have been summarized.

Steps in NGS workflow	QC Metric	Method/Indicator	Comments
**Nucleic Acid Quantification**	Nucleic acid quality and quantity	Fluorimetric dye-based or qPCR based quantification	Estimation of nucleic acids by UV absorption is not recommended as it has limited reflection on quality and is prone to interference by contaminants Fluorimetric dye-binding estimation provides better quantitation of intact double-stranded DNA or single stranded RNA as required qPCR-based methods provide an accurate measure of amplifiable nucleic acids
**Genomic library Preparation**	Genomic library quality and quantity	Gel-based systems or qPCR based quantification	An optimum yield of genomic library is an indicator of successful target enrichment and library preparation A minimum library concentration has to be defined which indicated successful library preparation for each sample
**Sequencing**	Run and sample level sequencing output and quality	**Sequencing output**—Number of bases and reads. Quality of base calling **Sequencing Quality**—sequencing accuracy and error rates. Average read length	Optimal sequencing output and quality has to be established for each platform and used as metrics to follow the performance of the sequencer Sequencing quality can be measured by metrics like - Phred quality scores—A score of Q30 would means 1 base calling error out of 1000 bases - Aligned quality (AQ) scores—A score of AQ20 means 1 misaligned base per 100 bases The minimum number of sequencing reads has to be defined per sample to ensure optimum sequencing The per sample read cutoff depends on the size of the gene panel and the desired sequencing depth
**Variant detection and clinical reporting**	Variant detection and reporting confidence	Presence of variant at optimal allelic frequency and sequencing depth Visual inspection of the sequencing reads to confirm the presence of the variant in both forward and reverse strands	A positive control with known mutation is included in every sequencing run and used as a control for sequencing quality and variant calling accuracy Identifying true mutation calls in the background of sequencing artifacts and false positive calls using criteria like established-limit-of detection, minimum sequencing depth, allelic frequency and manifestation of mutation in both forward and reverse reads is critical prior to clinical reporting

In NGS tests that interrogate relatively large genomic areas in tumors (panels with hundreds of genes, whole exome or genome), it is recommended to sequence paired-normal DNA to facilitate better identification of the somatic driver mutations in the background of germ line variants. The source of normal DNA could be tumor-adjacent normal tissue, peripheral blood (lymphocytes), buccal swabs or saliva. A comparison of the mutation calls from the tumor to the paired normal will help in filtering the germ line variant background and better identification of the somatic driver mutations. Although optimal, the use of paired normal reference is challenging for several reasons, such as the increased cost of sequencing the normal reference and the lack of its timely availability for every tumor sample.

Once the true variants are identified, they must be annotated and reported in a comprehensible manner for the information to be used in clinical decision-making. To facilitate this, the variants need to be clearly annotated with regard to the gene name (as per HUGO nomenclature guidelines) [42], the type of change in the coding sequence, and the resulting change in the protein. The annotation guidelines for the variants have been defined by the Human Genome Variation Society (HGVS) [43]. In addition to the annotated variants, the clinical reports must also clearly list the genes and the areas of the genes being tested (exons and codons). The areas that are intended to be tested but are not captured by the target enrichment method (failed regions) also need to be listed. The purpose of the NGS test and its limitations regarding detection of different varieties of mutations need to be clearly defined. The report must also list the reference genome, versions of the analytical software and the public variant databases (COSMIC, dbSNP, ClinVar, *etc.*) that are used for annotation. In addition to annotation, it is also recommended that the functional and clinical significance of the variants be stated in the clinical report. This can be done by referring to a well-annotated database of reported somatic mutations such as COSMIC, My Cancer Genome database from Vanderbilt University, the Human Gene Mutation Database (HGMD), and the DNA-mutation Inventory to Refine and Enhance Cancer Treatment (DIRECT) database, which record the occurrence frequencies, biological and clinical significance of mutations in cancers [44,45,46].

NGS testing includes relatively large genomic areas, which results in the discovery of genomic aberrations other than those with direct clinical relation to the disease. These aberrations could have clinical implications and are referred to as *secondary* or *incidental findings.* They are controversial regarding their disclosure to the patient and associated legal and ethical implications. Recently, policy statements from two major regulatory agencies (AMP and ACMG) have provided much-needed clarity on this issue [47,48]. Guidelines from ACMG stress on the importance of discussing the possibility of incidental findings with the patient before the test and state that all incidental findings need to be reported for constitutional NGS testing (exome and genome) including the normal from the normal-tumor sets. Reporting was deemed mandatory for a recommended set of 56 genes for subjects of all ages with the exception of fetal samples [47]. Similarly, the report from AMP also stressed the importance of obtaining consent from the patient before the test, and the recommendations included reporting only pathogenic and likely pathogenic variants in a defined set of genes clearly stated in the report. Confirmation of the reported pathogenic variants by an orthogonal technique was recommended, along with submission of the confirmed incidental findings to public databases such as ClinVar and Leiden Open databases [48].

An additional challenge to the implementation of routine clinical NGS in diagnostic laboratories is the reimbursement policies by the insurance companies or payers. These policies are well-suited toward single gene tests and are yet to evolve to meet the challenges and complexities of multiple marker NGS testing. A recent review provides an excellent detailed account of the reimbursement policies of public and private payers, their consequences on clinical NGS, and the measures being taken to alleviate them [49].

## 4. Potential for the Future

A general trend observed in all molecular technologies is the natural evolution of platforms toward greater levels of multiplexing involving screening of multiple markers in multiple samples. Due to these obvious advantages, the improved technologies are desirable but are associated with challenges regarding the performance evaluation and interpretation of higher-complexity data. This trend has characterized the onset and application of the revolutionary NGS technologies superseding the traditional low- and medium-throughput sequencing technologies. In terms of mutation screening of tumors, the choice of NGS over the traditional orthogonal sequencing technologies both for research purposes and clinical diagnostics is justified due to its associated advantages. The value of NGS for routine diagnostics is bound to grow in light of high discovery rate of new markers. This will warrant routine screening of multiple markers, more often than not from a limited amount of nucleic acids of varying quality.

**Figure 1:**
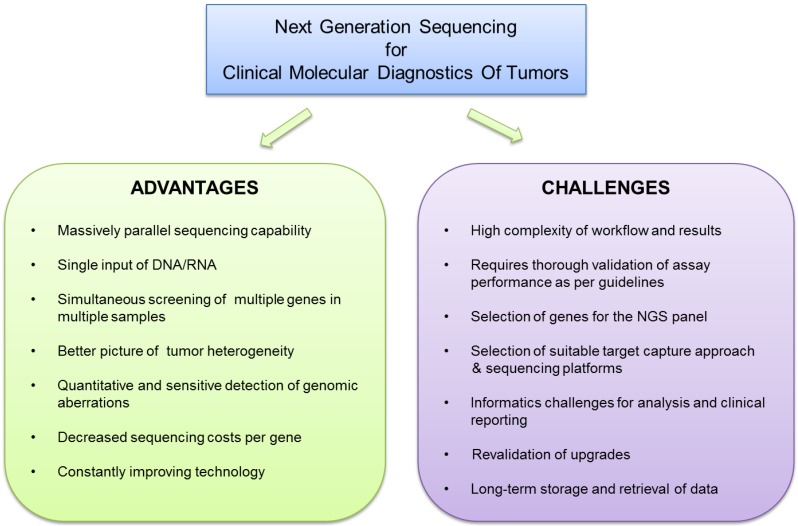
Advantages and challenges of clinical NGS: The advantages and challenges associated with implementation of next-generation sequencing technologies in a clinical molecular diagnostic laboratory have been summarized.

In recent years, we have witnessed rapid improvements in target capture, library preparation and sequencing solutions that have provided the impetus for increased acceptance and implementation of NGS. In addition, several studies that have compared in-detail every aspect of NGS technology, such as wet bench processes and platforms, informatics for data analysis and costs for testing have brought greater clarity with respect to choosing the right options. Furthermore, recommendations and guidelines drafted by several regulatory bodies have also provided clarity regarding the validation and implementation of NGS-based assays in a clinical diagnostic environment. The expected onset of novel sequencing technologies such as the Nanopore technology has the potential to vastly revolutionize parallel sequencing and contribute further towards establishing NGS as preferred clinical sequencing technologies. These novel technologies have the potential to complement and/or potentially replace the current NGS technologies.

To summarize, NGS technologies, which represent a major revolution in genome sequencing, are able to meet the challenges associated with the increased need for routine mutation profiling of tumors. However, the high complexity of this technology and performance validation poses distinct challenges for successful adaptation in the clinical diagnostic environment (summarized in Figure 1). Increased clarity regarding the validation and implementation of NGS tests by several regulatory agencies, published reports from several clinical laboratories and technological improvements have made the implementation of NGS technologies more feasible, thus establishing them as the most preferred large-scale genome sequencing technologies.

## Supplementary Files

Supplementary File 1
