# Supplementary material for: Next-Generation Sequencing in Clinical Molecular Diagnostics of Cancer: Advantages and Challenges
Source: Cancers (Basel). 2015 Oct 14;7(4):2023–36. doi: 10.3390/cancers7040874 (PMC4695874; doi:10.3390/cancers7040874)
Supplement: Supplementary File 1 [file cancers-07-00874-s001.pdf]

Supplementary Materials

Table S1. Comparison of direct costs for NGS-based test and traditional platforms. A comparison of direct costs (including reagents, personnel hands-on time and consumables costs) for commonly tested markers like *BRAF*, *NRAS*, *KIT*, *JAK2* as analyzed by traditional platforms and a NGS test are listed below.

Gene	Assay Technique	Direct Cost ($) *
BRAF	Pyrosequencing (Codon 600)	245.00
NRAS	Pyrosequencing (Codons 12,13 and 61)	261.13
KIT	Sanger Sequencing (Exons 9, 11, 13, 17)	419.35
***JAK2* (*p. V617F*)**	Sanger Sequencing	277.07
***JAK2* (*p. V617F*)**	Pyrosequencing	208.16
50 gene panel **(*including BRAF, NRAS, KIT, JAK2*)**	NGS (Ion Torrent PGM) 50-cancer related genes Hotspot mutations	574.20

* The direct costs include reagents, labor and consumables. They can vary -for each lab depending upon any discount rates extended by the vendor and personnel costs.

Table S2. Comparison of Analytical sensitivity of NGS to traditional sequencing and other NGS platforms. A comparison of the sensitivity of NGS platforms validated in our laboratory to other sequencing technologies as has been summarized.

NGS Panel	NGS Sensitivity Compared to				
	**Sanger Sequencing**	**Pyro Sequencing**	**Primer Extension-Mass Array**	**NGS**	**MIP [&] Array (for CNVs)**
46 gene ***(Ion Torrent-PGM)***	96% *	100%	100%	NA	NA
53 gene panel ***(MiSeq)***	100%	100%	NA	100% [#]	NA
409 gene panel ***(Ion Proton)***	100%	NA	NA	95% [$]	100%

* Missed large insertions and deletions due to variant caller issues. Fixed by upgraded version of the software; [#] As compared to 46 gene panel on Ion Torrent PGM; [$] As compared to 46 gene panel using Ion Torrent PGM. Missed calls were due to failed sequencing or lack of sequencing coverage in the 409 gene panel; [&] MIP Array (OnoScan Molecular Inversion Probe array, Affymetrix).
